# Sex-related differences in extracranial complications in patients with traumatic brain injury

**DOI:** 10.3389/fneur.2023.1095009

**Published:** 2023-04-06

**Authors:** Giovanna Brandi, Alma Gambon-Mair, Lara Selina Berther, Stefan Yu Bögli, Simone Unseld

**Affiliations:** ^1^Institute of Intensive Care Medicine, University Hospital Zurich, Zurich, Switzerland; ^2^Department of Neurology, University Hospital Zurich, Zurich, Switzerland; ^3^Clinical Neuroscience Center, University Hospital Zurich, Zurich, Switzerland

**Keywords:** traumatic brain injury, sex-specific, extracranial complications, systemic complications, intensive care, outcome

## Abstract

**Background:**

Extracranial complications after traumatic brain injury (TBI) are common. Their influence on outcome is uncertain. Furthermore, the role of sex on the development of extracranial complications following TBI remains poorly investigated. We aimed to investigate the incidence of extracranial complications after TBI with particular focus on sex-related differences with regard to complications and their influence on outcome.

**Methods:**

This retrospective, observational study was conducted in a level I universitary swiss trauma center. Consecutive patients with TBI admitted to the intensive care unit (ICU) between 2018 and 2021 were included. Patients’ and trauma characteristics, in-hospital complications (i.e., cardiovascular, respiratory, renal, metabolic, gastrointestinal, hematological, and infectious) as well as functional outcome 3 months after trauma were analyzed. Data was dichotomized by sex or by outcome. Univariate as well as multivariate logistic regression was performed to reveal possible associations between sex, outcome and complications.

**Results:**

Overall, 608 patients were included (male *n* = 447, 73.5%). Extracranial complications occurred most frequently in cardiovascular, renal, hematological and infectious systems. Men and women suffered similarly from extracranial complications. While men needed correction of coagulopathies more often (*p* = 0.029), women suffered more frequently from urogenital infections (*p* = 0.001). Similar results were found in a subgroup of patients (*n* = 193) with isolated TBI. A multivariate analysis did not show extracranial complications to be independent predictors of unfavorable outcome.

**Conclusion:**

Extracranial complications following TBI occur frequently during the ICU-stay, can affect almost all organ systems but are not independent predictors of unfavorable outcome. The results suggest that sex-specific strategies for early recognition of extracranial complications might not be needed in patients with TBI.

## 1. Introduction

Traumatic brain injury (TBI) is the leading cause of mortality in young adults and a major cause of death and disability across all ages and countries (1). Extracranial complications following TBI are frequent and are important contributors to morbidity and mortality (2). More than 50% of patients with TBI suffer from major extracranial complications (3). Some are directly associated to the massive catecholamine release and neuro-inflammatory response following TBI, some others arise during the intensive care unit (ICU) stay (4–6). Early recognition and prompt treatment of extracranial complications may improve overall outcome (5).

Sex-specific differences exist in almost all aspects of diseases. Risk profile, etiology and the course of a disease including morbidity and mortality can vary by sex (7). Particularly in young adulthood, males are at greater risk of TBI compared to females. Mechanisms of trauma differ by sex: while for young males motor vehicle accidents, sports-related injuries and increased interpersonal violence represent the most common causes of trauma (8, 9), women are more likely to sustain TBI due to falls and intimate partner/domestic violence. However, as cultural shifts continue and women are more often involved in higher-risk activities and military service, these differences may diminish (10, 11).

Considering outcome after TBI, the role of sex on outcome is not completely understood with conflicting published results. Generally, worse outcomes in women after mild TBI have been reported (12). On the contrary, functional outcome and mortality were mostly similar or better in women in moderate to severe TBI (13, 14).

The association of sex on the development of in-hospital extracranial complications following TBI remains poorly investigated.

This retrospective study investigates sex-specific extracranial complications during the ICU-stay in patients with TBI. The identification of sex-specific complications following TBI is of clinical relevance and of interest to evaluate and improve the current monitoring strategies, to offer a more personalized management, and to better distribute the available resources.

## 2. Materials and methods

### 2.1. Study design

This retrospective, observational, single-center study was conducted at the Institute for Intensive Care, University Hospital of Zurich, Switzerland between January 2018 and September 2021. All patients with TBI admitted consecutively to the ICU were eligible for inclusion in the study. TBI was defined as an alteration in brain function, or other evidence of brain pathology, caused by an external force (15). The study was approved by the local ethics committee. It was performed in accordance with the ethical standards as laid down in the 1964 Declaration of Helsinki and its later amendments.

### 2.2. Inclusion and exclusion criteria

We included all consecutive adult patients (>18 years old) admitted to our trauma ICU following TBI, regardless of severity. Patients were excluded if they were < 18 years old, if the time from trauma to admission to our hospital was greater than 24 h, and/or in case of patient’s written or documented oral refusal to have his/her data analyzed for research projects.

### 2.3. Patients’ management

All patients were treated based on an institutional protocol which is based on the Brain Trauma Foundation guidelines (16). Individual decisions were taken by the attending senior ICU physician together with the neurosurgeon or trauma surgeon in charge.

### 2.4. Data collection

Data were obtained from the electronic medical records (KISIM-TM; Cistec^®^ Zurich, Switzerland) and the electronic Patient Data Management System (MetaVision Suite; iMDsoft^®^, Tel Aviv, Israel). Study data were collected and managed using Research Electronic Data Capture (REDCap; Vanderbilt University, Nashville, United States) (17, 18).

Demographic data collected included age, sex, presence of comorbidities based on the Charlson Comorbidities Index (CCI) (19), previous medications, living situation (alone, with others at home, retirement home, nursing home, or others), support in everyday life (independent/ dependent), as well as Clinical Frailty Index (20). Risky behaviors, such as alcoholism, smoking, and use of illicit drugs, were collected.

Data regarding injury included Injury Severity Score (ISS) (21), Abbreviated Injury Scale (AIS) for head and neck, face, chest, abdomen, extremities and external (22) as well as the initial Glasgow Coma Scale Scores (GCS) (23).

Extracranial complications were collected in an organ-specific manner, as:

- *cardiovascular*: including acute myocardial injury/ infarction, new or worsening heart failure, Takotsubo Syndrome, severe arrhythmia, cardiogenic shock, cardiac arrest, need for circulatory support or antihypertensive therapy, periods of prolonged hyper-or hypotension, endocarditis, myocarditis, pericarditis;

- *pulmonary*: including Acute Respiratory Distress Syndrome (ARDS), need and duration of mechanical ventilation (expressed in days), pneumothorax, pleural effusion;

- *gastrointestinal*: including gastrointestinal bleeding, peptic ulcer, gastrointestinal tract perforation, paralytic ileus, abdominal compartment syndrome, mesenteric ischemia, acute or acute on chronic liver failure, acute pancreatitis, cholestatic injury;

- *renal and metabolic*: including acute kidney injury, need for renal replacement therapy, rhabdomyolysis, adrenal insufficiency, syndrome of inadequate (Antidiuretic hormone) ADH secretion, cerebral salt wasting, diabetes insipidus, non-thyroidal illness syndrome, hyperammonemia, hypo- or hyperglycemia;

- *hematological*: including coagulation disorder, bleeding, disseminated intravascular coagulation, catheter-related thrombosis, deep vein thrombosis, pulmonary embolism, hemorrhagic shock, thrombocytopenia, heparin-induced thrombocytopenia;

- *infectious*: catheter-related bloodstream infection, aspiration pneumonia, nosocomial pneumonia, ventilator-associated pneumonia, septic shock, bacteremia, fungaemia, viraemia;

- *iatrogenic complications*: including allergic and non-allergic drug side effects, and miscellaneous including decubitus or damage due to patient positioning.

Definitions of each complication are presented in the Supplementary material.

During the ICU stay, the maximum Sepsis-related Organ Failure Assessment Score (SOFA) (24) value was considered.

Outcome was assessed at 3 months after hospital trauma by using the Extended Glasgow Outcome Scale (GOSE) acquired during regular post-ICU follow-up or from the rehabilitation reports (25).

### 2.5. Statistical analysis

Statistical analysis was performed using SPSS version 26. Data was always dichotomized by sex (male vs. female) or outcome (favorable (GOSE 5–8) vs. unfavorable (GOSE 1–4)). Subgroup analysis was performed separately for patients suffering isolated TBI with no extracranial trauma sequelae. Descriptives are reported as counts/percentages, mean ± standard deviation (SD), or as median including the interquartile range (IQR) as appropriate. All continuous data were tested for normality using Shapiro–Wilk’s test. Univariate or multivariate logistic regression was used to find variables associated with either sex or unfavorable outcome. Multivariate analysis was performed based on the univariate analysis to correct results for the differences in clinical characteristics aside from complications. Respective odds ratios (OR) including 95% confidence intervals (95%-CI) are only shown for significant associations. The significance level was set at *p* < 0.05.

## 3. Results

### 3.1. Demographics, injury severity, outcome

Overall, 608 patients were included in the study, 193 of them presenting with isolated TBI (iTBI). The majority of them were male (73.5%). Demographics, living situation prior to TBI, Frailty index, comorbidities including CCI, risky behaviors, severity of injury and outcome are presented in Table 1 (including all patients) and Table 2 (including only patients with iTBI). Considering all patients, men were younger (mean 51 vs. 61 years), were more likely to be independent in daily living (91 vs. 83%), and less frail. Interestingly, alcoholism, smoking and illicit drug use occurred in similar frequencies in either sex. Both sexes were relatively healthy before TBI when considering the CCI. Injury characteristics and disease severity were similar when considering SOFA, ISS or initial GCS. However, women were more likely to suffer from more severe damage to the Head and Neck (median Head and Neck AIS 2 vs. 3). Notably, even when only considering iTBI, women were suffering from more severe damage to Head and Neck. There were no significant differences in trauma mechanism (Data not shown), but women suffered more often from accidental injury (68.8 vs. 58.9%). Outcome was worse in women.

**Table1:** Demographics dichotomized by sex and outcome (all Patients).

		Male (73.5)	Female (26.5)	*p*-value	OR	95%-CI		Outcome: Favorable (55.6)	Outcome: Unfavorable (44.4)	*p*-value	OR	95%-CI	
Sex (female)								21.6	32.6	0.002	1.755	1.221	2.524
Age		51 ± 24.8	61 ± 21.8	< 0.001	1.019	1.01	1.028	48 ± 24.3	61 ± 22.6	< 0.001	1.025	1.017	1.033
Living situation	**Alone at home**	24.4	32.3					27.5	25.2				
	**With other persons at home**	61.1	49.7					58.9	57.0				
Support in everyday life	**Independent**	91.1	82.6	0.014	1.716	1.118	2.635	95.6	80.4	0.001	3.392	2.032	5.663
Estimated clinical Frailty Index	**Very fit**	25.5	15.5	< 0.001	1.347	1.163	1.56	25.7	19.3	< 0.001	1.492	1.296	1.719
	**Well**	38.9	34.2					46.2	27.0				
	**Managing well**	18.3	20.5					13.0	26.3				
	**Vulnerable**	12.8	19.3					12.7	16.7				
	**Mildly frail**	3.6	8.7					2.4	8.1				
	**Moderately to severely frail**	0.9	1.8					0.0	2,6				
Alcoholism		19.2	18.0	0.733				21.0	16.3	0.142			
Smoking		16.8	18.6	0.549				18.6	15.6	0.318			
Illicit drug use		9.2	5.6	0.16				7.7	8.9	0.594			
Charlson comorbdity index		0 [0, 1]	0 [0, 1]	0.903				0 [0, 1]	0 [0, 2]	< 0.001	1.273	1.137	1.425
SOFA		7 [4, 10]	7 [5, 10]	0.719				5 [3, 8]	10 [8, 12]	< 0.001	1.383	1.305	1.465
Non-head ISS		10 [2, 18]	8 [1, 16.5]	0.315				9 [2, 18]	8.5 [1, 18]	0.291			
ISS		22 [14, 29]	25 [15, 30]	0.171				17 [13, 26]	26 [21, 38]	< 0.001	1.74	1.0561	1.092
AIS	**Head and Neck**	2 [2, 4]	3 [3, 5]	0.043				3 [2, 3]	4 [3, 5]	< 0.001	1.953	1.692	2.255
	**Facial**	0 [0, 2]	0 [0, 1.5]	0.087				0 [0, 2]	0 [0, 2]	0.61			
	**Chest**	0 [0, 3]	0 [0, 3]	0.258				0 [0, 3]	0 [0, 3]	0.579			
	**Abdomen**	0 [0, 1]	0 [0, 2]	0.772				0 [0, 1]	0 [0, 2]	0.564			
	**Extremity, pelvis**	0 [0, 2]	0 [0, 2]	0.297				0 [0, 2]	0 [0, 2]	0.956			
	**Other**	0 [0, 1]	0 [0, 1]	0.075				0 [0, 1]	0 [0, 1]	0.236			
Isolated TBI		31.5	32.3	0.86				31.1	32.6	0.688			
Initial GCS		13 [7, 15]	13 [6, 14]	0.132				14 [10, 15]	9 [3, 14]	<0.001	0.863	0.831	0.896
3-month GOSE	**1**	19.2	30.4	0.005	0.886	0.813	0.965						
	**2**	0.4	0.0										
	**3**	8.9	5.6										
	**4**	12.1	18.6										
	**5**	30.6	24.8										
	**6**	13.6	8.1										
	**7**	9.6	7.5										
	**8**	5.4	5.0										

Data shown as percentage, mean ± SD, or median [IQR] as appropriate. Univariate logistic regression was used to ascertain possible associations with either sex of unfavorable outcome. OR, odds ratio; CI, confidence interval; SOFA, sepsis-related organ failure assessment score; ISS, Injury Severity Score; TBI, traumatic brain injury; GCS, Glasgow Coma Scale; GOSE, Glasgow Outcome Scale Extended; AIS, Abbreviated Injury Scale.

**Table 2:** Demographics dichotomized by sex and outcome (TBI only).

		Male (73.5)	Female (26.5)	*p*-value	OR	95%-CI		Outcome: Favorable (55.6)	Outcome: Unfavorable (44.4)	*p*-value	OR	95%-CI	
Sex (female)								17.1	38.6	0.001	3.043	1.566	5.915
Age		55 ± 22.5	68 ± 19.8	0.001	1.029	1.012	1.046	52 ± 21.8	67 ± 20.7	0.001	1.032	1.017	1.047
Living situation	**Alone at home**	27.7	30.8	0.517				30.5	34.6	0.881			
	**With other persons at home**	56.0	51.9					56.2	63.9				
Support in everyday life	**Independent**	90.8	73.1	0.003	3.277	1.49	7.207	94.3	76.1	0.003	3.79	1.561	9.203
Estimated clinical Frailty index	**Very fit**	16.3	13.5	0.022	1.343	1.044	1.728	19.0	19.3	0.001	1.628	1.262	2.101
	**Well**	39.7	32.7					47.6	54.1				
	**Managing well**	23.4	15.4					14.3	16.2				
	**Vulnerable**	16.3	21.2					17.1	19.5				
	**Mildly frail**	2.8	13.5					1.9	2.2				
	**Moderately to severely frail**	0.7	3.8					0.0	0.0				
Alcoholism		28.4	17.3	0.121				27.6	31.4	0.437			
Smoking		15.6	13.5	0.712				14.3	16.2	0.753			
Illicit drug use		8.5	5.8	0.513				8.6	9.7	0.651			
Charlson comorbidity index		0 [0, 2]	0 [0, 1]	0.686				0 [0, 1]	1 [0, 2]	0.002	1.37	1.123	1.672
SOFA		6 [3, 10]	8 [4, 10]	0.782				5 [2, 7]	9 [7, 11]	0.001	1.425	1.282	1.585
ISS		17 [10, 25]	19 [14, 26]	0.266				14 [9, 19.5]	25 [17, 26.75]	0.001	1.129	1.08	1.18
AIS	**Head and Neck**	3 [3, 5]	4 [3, 5]	0.009	1.437	1.093	1.889	3 [3, 4]	5 [3.25, 5]	0.001	2.476	1.821	3.368
	**Facial**	0 [0, 2]	0 [0, 1]	0.041	0.706	0.506	0.986	0 [0, 2]	0 [0, 1]	0.095			
Initial GCS		13 [6, 15]	8 [4, 14]	0.042	0.933	0.873	0.998	14 [10, 15]	7 [3, 13]	0.001	0.829	0.775	0.887
3-month GOSE	**1**	22.0	48.1	0.001	0.791	0.687	0.91						
	**2**	0.0	0.0										
	**3**	6.4	5.8										
	**4**	9.9	11.5										
	**5**	24.1	11.5										
	**6**	15.6	5.8										
	**7**	14.9	15.4										
	**8**	7.1	1.9										

Data shown as percentage, mean ± SD, or median [IQR] as appropriate. Univariate logistic regression was used to ascertain possible associations with either sex of unfavorable outcome. OR, odds ratio; CI, confidence interval; SOFA, sepsis-related organ failure assessment score; ISS, Injury Severity Score; TBI, traumatic brain injury; GCS, Glasgow Coma Scale; GOSE, Glasgow Outcome Scale Extended; AIS, Abbreviated Injury Scale.

### 3.2. Complications

Table 3 shows the extracranial complications considering all patients, while Table 4 shows the extracranial complications only including iTBI. No major differences could be found between sexes for both groups with only males suffering from more coagulation disorders needing substitution (21.7 vs. 13.7%) and more women suffering from urogenital infections (0.4 vs. 5.6%) when considering all patients and women suffering from more infectious complications when considering iTBI (18.4 vs. 38.5%).

**Table 3:** Complications dichotomized by sex and outcome (all).

	All	Sex			Outcome:	Outcome:	
		Male (73.5)	Female (26.5)	OR (95%-CI)	Favorable (55.6)	Unfavorable (44.4)	OR (95%-CI)
*Cardiovascular, any*	62.3	60.9	66.5		50.0	77.8	3.50 (2.45–5.00)
Myocardial infarction	3.3	3.1	3.7		2.7	4.1	
Acute myocardial injury	37.8	38.5	36.0		27.8	50.4	2.63 (1.88–3.69)
Takotsubo syndrome	0.5	0.4	0.6		0.3	0.7	
Heart failure (new or worsening)	2.5	2.7	1.9		0.9	4.4	5.19 (1.45–18.60)
Severe arrhythmias	6.4	6.0	7.5		3.0	10.7	3.95 (1.89–8.25)
Prolonged hypotension	32.4	30.6	37.3		28.4	37.4	1.51 (1.07–2.12)
Prolonged hypertension	16.3	16.1	16.8		9.5	24.8	3.16 (2.00–4.99)
*Need for circulatory support, any*	69.4	68.2	72.7		57.7	84.1	3.87 (2.62–5.72)
*Norepinephrine*	69.1	67.8	72.7		57.1	84.1	3.97 (2.68–5.86)
*Epinephrine*	2.3	1.8	3.7		0.3	4.8	17.04 (2.22–131.15)
*Vasopressin*	9.9	11.2	6.2		2.7	18.9	8.51 (4.11–17.65)
*Dobutamine*	3.6	3.8	3.1		1.2	6.7	5.96 (1.99–17.84)
*ECMO*	0.2	0.2	0.0		0.0	0.4	
*Pulmonary, any*	3.8	3.8	3.7		3.3	4.4	
ARDS	0.5	0.2	1.2		0.6	0.4	
Pneumothorax (not iatrogenic)	0.7	0.4	1.2		0.3	1.1	
Pleural effusion worthy of puncture	1.2	0.9	1.9		0.3	2.2	
Pulmonary edema	0.2	0.0	0.6		0.0	0.4	
*Need for mechanical ventilation*	68.8	68.0	70.8		55.9	84.8	4.48 (3.01–6.68)
*Re-intubation*	8.6	8.3	9.3		5.9	11.9	
*Tracheotomy*	7.2	7.8	5.6		2.1	13.7	5.01 (2.18–11.52)
*Gastrointestinal, any*	29.4	28.6	31.7		22.8	37.8	2.06 (1.45–2.93)
GI bleeding	1.0	1.1	0.6		0.0	2.2	
Peptic ulcer	1.2	1.3	0.6		0.0	2.6	
Paralytic ileus	8.6	9.4	6.2		4.7	13.3	3.10 (1.68–5.71)
Abdominal compartment syndrome	1.2	1.3	0.6		0.6	1.9	
Acute pancreatitis	1.0	0.9	1.2		0.9	1.1	
Transaminitis	21.9	21.3	23.6		18.6	25.9	1.53 (1.04–2.25)
Acute cholestatic injury	5.4	4.5	8.1		2.4	9.3	4.21 (1.87–9.49)
Refeeding syndrome	3.0	2.9	3.1		2.4	3.7	
*Need for jejunal/duodenal feeding tube*	15.0	15.2	14.3		12.1	18.5	1.65 (1.05–2.58)
*Renal/metabolic, any*	47.4	46.1	50.9		40.5	55.9	1.86 (1.35–2.57)
Acute kidney injury	9.7	9.4	10.6		4.7	15.9	3.81 (2.10–6.94)
Rhabdomyolysis	3.9	4.7	1.9		2.1	6.3	3.18 (1.30–7.78)
Sodium disorders (endocrine-induced)	4.3	3.6	6.2		2.4	6.7	2.95 (1.26–6.89)
Euthyroid sick syndrome	2.8	3.1	1.9		2.7	3.0	
Hypoglycemia	20.7	19.7	23.6		20.1	21.5	
Hyperglycemia	21.4	20.4	24.2		15.1	29.3	2.33 (1.57–3.46)
Hyponatremia (excl. Endocrine disorder)	4.1	4.3	3.7		3.0	5.6	
Hypernatremia (excl. Endocrine disorder)	3.3	3.8	1.9		2.4	4.4	
*Need for renal replacement therapy*	2.0	2.5	0.6		1.2	3.0	
*Hematological, any*	33.9	33.6	34.8		24.0	46.3	2.74 (1.94–3.87)
Coagulation disorder needing substitution	19.6	21.7	13.7	0.57 (0.35–0.94)	13.3	27.4	2.46 (1.63–3.71)
Bleeding needing RBC transfusion	16.8	15.4	20.5		8.6	27.0	3.95 (2.48–6.29)
Hemorrhagic shock	4.9	4.9	5.0		1.5	9.3	6.80 (2.57–18.0)
Thrombozytopenia	9.5	9.4	9.9		7.4	12.2	1.74 (1.01–3.01)
*Infectious, any*	25.3	23.7	29.8		13.0	40.7	4.59 (3.09–6.85)
Catheter-related bloodstream infection	0.7	0.9	0.0		0.0	1.5	
Aspiration pneumonia	9.9	9.2	11.8		3.6	17.8	5.87 (3.05–11.31)
Nosocomial pneumonia	3.1	3.4	2.5		2.1	4.4	
Ventilator-associated pneumonia	7.1	6.9	7.5		3.0	12.2	4.57 (2.21–9.45)
Urogenital infection, urosepsis	1.8	0.4	5.6	13.17 (2.82–61.65)	1.2	2.6	
Gastrointestinal infection	0.2	0.2	0.0		0.3	0.0	
Wound infection, surgical site infection	2.0	2.2	1.2		1.2	3.0	
Septic shock	3.5	3.6	3.1		2.1	5.2	2.59 (1.03–6.50)
Bacteremia	2.5	3.1	0.6		1.2	4.1	3.55 (1.12–11.27)
Fungemia	0.3	0.4	0.0		0.0	0.7	
Viral infection	0.3	0.2	0.6		0.0	0.7	
*Others*	3.9	4.3	3.1		2.1	6.3	3.18 (1.30–7.78)
Other newly diagnosed on ICU (any)	8.9	8.7	9.3		7.7	10.4	
Drug side effects (non-allergic)	3.3	3.1	3.7		3.3	3.3	
Allergic drug reaction	1.3	1.3	1.2		0.9	1.9	

Data shown as percentage. Univariate logistic regression was used to ascertain possible associations with either sex of unfavorable outcome. ICU, Intensive Care Unit; ECMO, Extracorporeal membrane oxygenation; ARDS, acute respiratory distress syndrome; RBC, red blood cell. Data shown in italics were not added to the analysis considering the whole group as they were interventions and not complications *per se*.

**Table 4:** Complications dichotomized by sex and outcome (iTBI only).

	Sex			Outcome:	Outcome:	
	Male (73.1)	Female (26.9)	OR (95%-CI)	Favorable (54.4)	Unfavorable (45.6)	OR (95%-CI)
*Cardiovascular, any*	54.6	53.8		39.0	44.4	4.16 (2.26–7.67)
Myocardial infarction	3.5	5.8		2.9	3.2	
Acute myocardial injury	29.8	23.1		18.1	20.6	2.99 (1.55–5.76)
Takotsubo syndrome	1.4	0.0		1.0	1.1	
Heart failure (new or worsening)	2.8	3.8		1.9	2.2	
Severe arrhythmias	4.3	11.5		3.8	4.3	
Prolonged hypotension	21.3	21.2		16.2	18.4	
Prolonged hypertension	16.3	21.2		7.6	8.7	5.09 (2.16–11.95)
*Need for circulatory support, any*	61.7	65.4		49.5	56.3	3.70 (1.69–6.99)
*Norepinephrine*	61.0	65.4		48.6	55.2	3.58 (2.04–7.26)
*Epinephrine*	0.0	1.9		0.0	0.0	
*Vasopressin*	5.7	5.8		1.9	2.2	5.86 (1.23–27.92)
*Dobutamine*	2.8	1.9		1.9	2.2	
*ECMO*	0.0	0.0		0.0	0.0	
*Pulmonary, any*	2.1	3.8		2.9	3.2	
ARDS	0.7	0.0		0.0	0.0	
Pneumothorax (not iatrogenic)	0.0	1.9		0.0	0.0	
Pleural effusion worthy of puncture	0.0	3.8		1.0	1.1	
Pulmonary edema	0.0	1.9		0.0	0.0	
*Need for mechanical ventilation*	63.8	73.1		54.3	61.7	3.66 (1.88–7.12)
*Re-intubation*	5.0	9.6		2.9	3.2	
*Tracheotomy*	7.1	3.8		1.0	1.1	10.27 (1.28–82.12)
*Gastrointestinal, any*	18.4	19.2		10.5	11.9	3.39 (1.56–7.38)
GI bleeding	0.7	1.9		0.0	0.0	
Peptic ulcer	0.7	0.0		0.0	0.0	
Paralytic ileus	8.5	5.8		5.7	6.5	
Abdominal compartment syndrome	0.7	0.0		0.0	0.0	
Acute pancreatitis	0.0	1.9		0.0	0.0	
Transaminitis	10.6	13.5		6.7	7.6	2.88 (1.12–7.42)
Acute cholestatic injury	2.1	7.7		0.0	0.0	
Refeeding syndrome	1.4	0.0		1.0	1.1	
*Need for jejunal/duodenal feeding tube*	10.6	9.6		6.7	7.6	
*Renal/metabolic, any*	39.0	46.2		34.3	39.0	1.83 (1.03–3.28)
Acute kidney injury	8.5	9.6		2.9	3.2	6.43 (1.78–23.19)
Rhabdomyolysis	1.4	1.9		1.0	1.1	
Sodium disorders (endocrine-induced)	5.0	3.8		1.9	2.2	
Euthyroid sick syndrome	4.3	1.9		4.8	5.4	
Hypoglycemia	13.5	17.3		16.2	18.4	
Hyperglycemia	18.4	19.2		11.4	13.0	2.91 (1.36–6.23)
Hyponatremia (excl. Endocrine disorder)	6.4	7.7		5.7	6.5	
Hypernatremia (excl. Endocrine disorder)	2.1	1.9		1.0	1.1	
*Need for renal replacement therapy*	1.4	0.0		1.0	1.1	
*Hematological, any*	24.1	13.5		14.3	16.2	2.51 (1.23–5.13)
Coagulation disorder needing substitution	14.2	5.8		7.6	8.7	2.49 (1.01–6.19)
Bleeding needing RBC transfusion	7.8	1.9		1.0	1.1	14.86 (1.88–117.53)
Hemorrhagic shock	2.1	1.9		0.0	0.0	
Thrombozytopenia	5.0	1.9		3.8	4.3	
*Infectious, any*	18.4	38.5	2.76 (1.37–5.58)	8.6	9.7	5.28 (2.57–10.85)
Catheter-related bloodstream infection	0.7	0.0		0.0	0.0	
Aspiration pneumonia	11.3	17.3		3.8	4.3	7.91 (2.60–24.09)
Nosocomial pneumonia	0.7	3.8		0.0	0.0	
Ventilator-associated pneumonia	5.0	5.8		1.9	2.2	5.15 (1.06–24.92)
Urogenital infection, urosepsis	0.0	7.7		0.0	0.0	
Gastrointestinal infection	0.0	0.0		0.0	0.0	
Wound infection, surgical site infection	0.7	1.9		1.0	1.1	
Septic shock	2.1	3.8		1.0	1.1	
Bacteremia	2.8	1.9		0.0	0.0	
Viral infection	0.7	0.0		0.0	0.0	
*Others*	2.8	3.8		1.9	2.2	
Other newly diagnosed on ICU, any	5.0	3.8		2.9	3.2	
Drug side effects (non-allergic)	0.7	0.0		1.0	1.1	
Allergic drug reaction	1.4	0.0		1.0	1.1	

Data shown as percentage. Univariate logistic regression was used to ascertain possible associations with either sex of unfavorable outcome. ICU, Intensive Care Unit; ECMO, Extracorporeal membrane oxygenation; ARDS, acute respiratory distress syndrome; RBC, red blood cell; HIT, Heparin-induced thrombocytopenia. Data shown in italics were not added to the analysis considering the whole group as they were interventions and not complications *per se*.

Unfavorable outcome on the other hand was associated with various known predictors including age, prior support in everyday life, frailty, CCI, ISS, AIS, and initial GCS. Frequent and clinically relevant complications (acute myocardial injury, new or worsening heart failure, severe arrhythmias, prolonged hypo- or hypertension; paralytic ileus, transaminitis, acute cholestatic injury; acute kidney injury, sodium disturbances, hyperglycemia; coagulation disorders needing substitution, bleeding needing RBC transfusion, hemorrhagic shock, thrombocytopenia; ventilator-associated pneumonia, aspiration pneumonia, ventilator-associated pneumonia, urogenital infection, septic shock and bactereremia) and associated interventions (administration of norepinephrine, epinephrine, vasopressin or dobutamine, mechanical ventilation, tracheotomy, need for jejunal/duodenal feeding tube) were associated to unfavorable outcome.

Due to the differences in presentation as mentioned above, a multivariate analysis was performed to assess independence of the associated factors. Multivariate analysis considering all patients and their associations to outcome for each group of complications (each group of complications was added separately) was performed correcting the results for sex, age, frailty, CCI, SOFA, initial GCS, and ISS. None of the clinically relevant groups of complications proved to be independently associated with sex or outcome. Similar results were achieved when evaluating associations to sex and when considering only iTBI. Sex only remained independently associated with infectious complications after correction for age, frailty, ISS, initial GCS (OR 2.25, 95%-CI 1.01–5.03). For ease of reading the insignificant data of the multivariate analysis is not shown.

## 4. Discussion

In the present study, we investigated frequency of extracranial complications after TBI with particular focus on sex-related differences, and their influence on outcome.

Overall, we found that extracranial complications occur frequently during the ICU stay. Cardiovascular, renal, hematological, and infectious complications are the most frequent, not only considering the entire study population, but also in the subgroup of patients with iTBI. Most of the patients needed mechanical ventilation following acute respiratory failure. The reason for the acute respiratory failure was the neurological impairment or the need of sedation in most patients. In only approximately 20% of patients a disease affecting primarily the lungs (aspiration pneumonia, VAP, HAP, ARDS, and thorax trauma) was the reason for intubation and mechanical ventilation. These findings are in line with previous reports (2, 3, 26).

The inflammatory response following TBI leads to catecholamine storm with influences on almost all organ systems. To evaluate the influence of the extracranial complications on outcome, we conducted a multivariate analysis with unfavorable outcome as dependent variable. After correction for sex, age, Frailty score, comorbidities, SOFA, ISS, and initial GCS, we found that the extracranial complications were not predictors of unfavorable outcome, as previously reported (3). This finding is surprising. Extracranial complications could exacerbate the intracranial complications. Their effect on outcome could be indirect; unfortunately, with the available data we are not able to prove this possibility in our study population. Nevertheless, extracranial complications should be monitored and recognized as early as possible, because they can develop at varying timepoints throughout the course of the condition.

Analyzing sex-specific differences, we found that women were older than men at the time of trauma, and men were less frequently in need of support in everyday life prior to TBI. Presence of comorbidities, as assessed by the CCI, as well as risky behaviors (i.e., smoking, alcohol abuse, and use of illicit drugs) were similar among men and women. This last finding suggests a cultural shift in the population. Previously, more risky behaviors were reported for men than for women (27–29).

Overall, the extracranial complications occurred in similar frequencies in men and women, as previously described (30). In general, both women and men suffered from hematological complications with similar frequency, but more men than women needed the correction of coagulopathies necessitating the administration of coagulation factor concentrates, fresh-frozen-plasma and/ or platelet concentrates. Considering that this difference is not seen any more in the subgroup of patients with iTBI, it is reasonable to assume that the need of more intense correction of the coagulation in men might be associated to injuries other than only TBI.

In general, no differences by sex were found in frequency of infections. Urogenital infections were rare, overall, but more women than men suffered from it. Anatomical differences and the use of bladder catheters might be the reasons for more urogenital infections in women (31).

Contradicting prior reports presenting trauma patients, we did not observe that male sex was associated with pneumonia and sepsis (32–36). Possible reasons for these discrepancies are differences in study design and that the patient populations included in the previous studies are quite heterogeneous with potential confounding effects due to the severity of injuries other than TBI. Furthermore, at our ICU, patients with TBI are treated following an internal protocol, which might limit the occurrence of some complications during the ICU stay.

In patients with iTBI, it has been reported that male patients more often suffer from respiratory complications, digestive complications, infections, and urinary complications (30). Our study population is relatively young and it is possible that some relevant complications for the aging population (>65 years of age) are thus not detected in this study.

### 4.1. Strength and limitations of the study

The strength of this study lies in the use of a database of consecutive patients with TBI during a 4-year period with various parameters collected and few missing data. Furthermore, we present an extensive list of complications including 7 major groups and over 80 possible distinct complications, which can help critical care physicians to consider them. In addition, very few studies investigated differences in kind and frequency of extracranial complications following TBI stratified by sex. However, this study has also several limitations: firstly, this is a retrospective single-center study, originating from a country with high quality of services and resources availability. This limits the generalizability of our findings. Secondly, while most patients with TBI are admitted to the ICU in our institution, some patients with mild TBI may have also been admitted to the in-house intermediate care unit and thus, not been included in the analysis. Thirdly, pre-admission status could only be recorded in a retrospective manner bearing the risk of under- or overestimation of the functional status of the patients prior to TBI. Fourthly, we did not find a direct effect of the extracranial complications on unfavorable outcome, but with the available data, we cannot exclude an indirect effect. Fourthly, functional outcome was evaluated only at 3 months after TBI, excluding the possibility of a longterm effect. Fifthly, the majority of patients suffered from a polytrauma, but the same results were found considering only patients with isolated TBI. Finally, the group size between sexes is skewed with much higher number of men and the women included were older. This, however, is a representation of the TBI cohort around Zurich. We believe that rather than comparing matched groups, we aimed to provide a detailed description of the whole group providing more information for day-to-day use instead of an evaluation whether sex matched for the same TBI characteristics and demographics would be a predictor of extracranial complications.

## 5. Conclusion

Following TBI, extracranial complications during the ICU stay occur frequently and affect all organ systems, but in our cohort they were not independent predictors of unfavorable outcome.

From a practical point of view, these findings suggest that sex-specific strategies might not be necessary for the early recognition of extracranial complications at the ICU in patients with TBI.

## Data availability statement

The raw data supporting the conclusions of this article will be made available by the authors, without undue reservation.

## Ethics statement

The studies involving human participants were reviewed and approved by Kantonale Ethikkommission Kanton Zürich. The patients/participants provided their written informed consent to participate in this study.

## Author contributions

GB and SB conceived the study, designed the study, interpretated the data, and drafted the article. AG-M designed the study, acquired the data, and revised the manuscript for intellectual content. LB acquired the data. SU designed the study, supervised the data acquisition, interpreted the data, and revised the manuscript for intellectual content. GB, AG-M, SB, and SU approved the final version. All authors contributed to the article and approved the submitted version.

## Funding

This study did not receive any funding. The use of REDCap was made available by the Swiss Society of Intensive Care Medicine.

## Conflict of interest

The authors declare that the research was conducted in the absence of any commercial or financial relationships that could be construed as a potential conflict of interest.

## Publisher’s note

All claims expressed in this article are solely those of the authors and do not necessarily represent those of their affiliated organizations, or those of the publisher, the editors and the reviewers. Any product that may be evaluated in this article, or claim that may be made by its manufacturer, is not guaranteed or endorsed by the publisher.
