# Supplementary material for: Sex-related differences in extracranial complications in patients with traumatic brain injury
Source: Front Neurol. 2023 Apr 6;14:1095009. doi: 10.3389/fneur.2023.1095009 (PMC10155273; doi:10.3389/fneur.2023.1095009)
Supplement: Supplementary file 1 [file Table_1.docx]

| **Organ System** | **Complication** | **Definition** |
| --- | --- | --- |
| Cardiovascular | Acute myocardial injury | Elevation of troponin above the 99^th^ URL, with a newly detected rising and/or falling pattern [1] |
|  | Myocardial infarction | Rise and / or fall of troponin values with at least 1 value above the 99th percentile URL and with at least 1 of the following:   - Clinical symptoms of myocardial ischemia - New ischemic ECG-changes - Development of pathological Q-waves - New regional wall motion abnormality in a pattern consistent with ischemic etiology - Identification of a coronary thrombus by angiography including intracoronary imaging or by autopsy [1] |
|  | Takotsubo Syndrome | - Transient regional wall motion abnormalities of LV or RV myocardium, usually extending beyond a single epicardial vascular distribution and often result in circumferential dysfunction of the ventricular segments involved - Absence of culprit atherosclerotic coronary artery disease or other pathological conditions like hypertrophic cardiomyopathy, viral myocarditis - New and reversible ECG abnormalities [2] |
|  | Heart failure (new or worsening) | Symptoms and/or signs caused by a structural and/or functional cardiac abnormality (LVEF<50%, abnormal cardiac chamber enlargement, E/E′>15, moderate/severe ventricular hypertrophy or moderate/severe valvular obstructive or regurgitant lesion) and corroborated by at least one of the following:   - Elevated natriuretic peptide levels - Objective evidence of cardiogenic pulmonary or systemic congestion (X-ray, echocardiography, direct measurement with catheter) [3] |
|  | Severe arrhythmia | All arrhythmias needing intervention (hemodynamic instability leading to increased dosing of catecholamines, application of antiarrhythmic drugs or electric cardioversion),  detected through PDMS or mentioned in ICU discharge papers. |
|  | Cardiogenic shock | SBP <90 mmHg after adequate volume resuscitation and clinical or laboratory signs of hypoperfusion   - Clinical hypoperfusion: Cold extremities, oliguria, mental confusion, dizziness, narrow pulse pressure, - Laboratory hypoperfusion: Metabolic acidosis, elevated serum lactate, elevated serum creatinine [4] |
|  | Endocarditis | According to the modified Duke Criteria [5] |
|  | Myocarditis | Inflammation of the heart of recent onset (< 1 month), caused by infections, exposure to drugs or toxic substances, and abnormal [immunoreactivity](https://www.sciencedirect.com/topics/medicine-and-dentistry/immunoreactivity) [6] |
|  | Pericarditis | 2 of the following 4 criteria:   - characteristic chest pain - pericardial friction rub - suggestive ECG changes - new or worsening pericardial effusion [7] |
|  | Pericardial effusion | If hemodynamically relevant, mentioned in echocardiography reports or ICU discharge papers |
|  | Prolonged hypertension | SBP > 180mmHg or MAP > 120mmHg ≥10 minutes |
|  | Prolonged hypotension | SBP < 90mmHg of MAP < 65mmHg  ≥10 minutes |
|  | Cardiac arrest | Abrupt loss of cardiac function leading to cardiopulmonary resuscitation |
|  | Need for circulatory support | Administration of Norepinephrine, Epinephrine or Argipressine documented in PDMS |
|  | Need for enteral or parenteral antihypertensive therapy | Administration of Urapidil, Labetalol, ACE-inhibitors or Calcium-antagonist drugs documented in PDMS |
| Pulmonary | ARDS | According to the Berlin Definition 2012 [8] |
|  | Mechanical Ventilation | Date of intubation, exubation, tracheotomy and duration of invasive ventilation documented in PDMS (expressed in days) |
|  | Pneumothorax | Presence of air in the pleural cavity, detected through plain X-rays, CT scans or sonography; diagnosed during the ICU stay |
|  | Pleural effusion | Fluid in the pleural cavity, detected through plain X-rays, CT-Scans or sonography, only if paracentesis was performed |
| Gastrointestinal | Gastrointestinal bleeding | Hemorrhage occurring in the gastrointestinal tract, proven by endoscopy |
|  | Peptic ulcer | Upper gastrointestinal mucosal lesions found endoscopically [9] |
|  | Paralytic Ileus | Functional blockage of the small and/or large intestine resulting in elevated gastric residual volume (> 500ml in 6 hours) and administration of procinetic drugs (i.e. Metoclopramide, Erythromycin, Dexpanthenol or Neostigmine) [10] |
|  | Abdominal compartment syndrome | Sustained IAP > 20mmHg that is associated with new organ dysfunction / failure [11] |
|  | Mesenteric ischemia | Sudden interruption or reduction of the blood supply to a segment of the small intestine, resulting in an insufficiency to meet the metabolic demands of the visceral organs [12], diagnosed by surgical inspection or through imaging |
|  | Gastrointestinal tract perforation | Loss of continuity of the gastric or intestinal mucosal wall, diagnosed by surgical inspection or through imaging |
|  | Acute or acute on chronic liver failure | Severe liver injury, potentially reversible in nature and with onset of hepatic encephalopathy within 8 to 28 days of the first symptoms in the absence of pre-existing liver disease [13], clinically manifesting in hepatic dysfunction, abnormal liver biochemical values, and coagulopathy |
|  | Transaminitis | Alanin-aminotransferase ≥ 2x URL |
|  | Acute pancreatitis | Two of the following three features:   - abdominal pain consistent with acute pancreatitis - serum lipase activity (or amylase activity) at least three times greater than the URL - characteristic findings of acute pancreatitis on contrast-enhanced CT, less commonly MRI or transabdominal ultrasonography [14] |
|  | Cholestatic injury | Alkaline phosphatase ≥1.5 x URL and gamma-glutamyl transferase ≥ 3x URL [15] |
|  | Duodenal feeding tube | Feeding tube with the distal end being placed in the duodenum under endoscopic guidance, |
| Renal / metabolic complications | Acute kidney injury | Elevation of creatinine ≥1.5x over baseline or ≥ 26.5mmol/L increase in creatinine for 6-12h [16] |
|  | Renal replacement therapy | Continuous or intermittent hemodialysis, hemofiltration or hemodiafiltration |
|  | Rhabdomyolysis | Creatinin kinase ≥ 10'000U/L or ≥ 5-10 x URL [17] |
|  | Adrenal insufficiency | Random cortisol level < 275nmol/L or delta total serum cortisol < 250nmol/L 60 minutes after i.v.-application of 250mcg cosyntropin [18] or favourable response after empirical administration of hydrocortisone 100mg i.v. |
|  | Hyponatraemia | Na < 135mmol/L |
|  | Syndrome of inadequate ADH secretion | Diagnosis of exclusion, hyponatraemia associated with a serum hypoosmolality of less than 275 mOsm/kg H2O, urine osmolality > 100mOsm/kgH2O, urine sodium > 30mmol/L [19] |
|  | Cerebral salt wasting | Hyponatraemia with increased urinary sodium concentration and hypovolemia in the setting of acute intracranial disease [20] |
|  | Diabetes insipidus | Polyuria (> 3L/24h) and urine osmolality <300 mOsm/kg H2O, leading to hypernatraemia [21] |
|  | Non-thyroidal illness syndrome (“Euthyroid sick syndrome”) | Decreased serum concentrations of thyroid hormones (especially T3) without a concomitant rise in serum TSH in patients without known prior thyroidal illness [22] |
|  | Hyperammoniaemia | BUN ≥ 60 umol/L |
|  | Hypoglycaemia | Serum glucose < 5mmol/L [23] |
|  | Hyperglycaemia | Serum glucose > 11mmol/L [24] |
| Hematological | Coagulation disorder needing transfusion | Application of fibrinogen, tranexamic acid, factor XIII concentrate, fresh frozen plasma and/or platelets concentrates |
|  | Bleeding needing transfusion | Applications of erythrocyte or thrombocyte concentrates |
|  | Disseminated intravascular coagulation | According to the scoring system developed by the International Society on Thrombosis and Hemostasis (ISTH) [25] |
|  | Catheter-related thrombosis | Thrombosis associated with a central venous catheter, diagnosed through angio-CT-scan or Doppler-ultrasound |
|  | Deep vein thrombosis | Occlusion of a deep vein, diagnosed by compression ultrasonography or angio-CT-scan |
|  | Pulmonary embolism | Embolism in the pulmonary vasculature, detected through CT pulmonary angiography |
|  | Hemorrhagic shock | Massive transfusion (4 PRBCs in 1 hour and continuous need for transfusion) vasopressor support and signs of organ hypoperfusion (lactate ≥ 2mmol/L, oliguria ≤0.5ml/kg/h) [26] |
|  | Thrombocytopenia | Platelets count < 80G/L |
|  | Heparin-induced thrombocytopenia | HIT-antibodies, testing only in cases with high pretest probability according to the HIT4T-Score [27], IgG-antibody against PF4/heparin, chemoluminescence testing (HemosILAcuStar), cut off 1.0 U/ml |
| Infectious | Extracranial surgical site infection | Infection related to a surgical procedure that occurs near the surgical site within 30 days following surgery |
|  | Catheter-related bloodstream infection | Bacteriaemia originating from an intravenous catheter: positive blood cultures drawn from the catheter and proof of bacterial growth on said catheter after removal [30] |
|  | Aspiration pneumonia | Fever, sputum or impaired oxygenation, inflammatory findings on chest radiography and overt / strongly suspected aspiration |
|  | Nosocomial pneumonia | Pneumonia developing > 48 hours after hospital admission [31] |
|  | Ventilator-associated pneumonia | Hospital-acquired pneumonia developing after > 48 hours of ventilation [31] |
|  | Urogenital infection, Urosepsis | Positive cultures obtained from urine, urethral sample, vaginal sample |
|  | Gastrointestinal infection | Positive stool samples for enteropathogenes or documented infection with C. difficile (antigen testing in patients with diarrhea, confirmation of positive results using testing for C.difficile toxin) |
|  | Septic shock | Sepsis plus vasopressor requirement to maintain a mean arterial pressure of 65 mm Hg or greater and serum lactate level greater than 2 mmol/L in the absence of hypovolemia [32] |
|  | Bacteriaemia | Blood cultures showing bacterial growth |
|  | Fungaemia | Blood cultures showing fungal growth |
|  | Viraemia | Proof of viral replication on serum PCR testing |
| Iatrogenic | Adverse drug reaction | An appreciably harmful or unpleasant reaction, resulting from an intervention related to the use of a medicinal product, which predicts hazard from future administration and warrants prevention or specific treatment, or alteration of the dosage regimen, or withdrawal of the product [33] |
|  | Other ICU complications | Decubitus, damage due to patient positioning, compartment syndrome of extremities |

Abbreviations:

ADH, antidiuretic hormone. ARDS, acute respiratory distress syndrome. BUN, blood urea nitrogen. CSF, cerebrospinal fluid. CT, computed tomography. ECG, electrocardiography. EEG, electroencephalography. HIT, heparine-induced thrombocytopenia. IAP, intra-abdominal pressure. ICU, intensive care unit. LV, left ventricle. LVEF, left-ventricular ejection fraction. MAP, mean arterial pressure. MRI, magnetic resonance imaging. PBRC, packed red blood cells. PDMS, patient data management system. PF4, platelet factor 4. SBP, systolic blood pressure. T3, triiodothyronine. TSH, thyroid stimulating hormone. URL, upper reference limit.

1. Thygesen, K., et al., *Fourth Universal Definition of Myocardial Infarction (2018).* Circulation, 2018. **138**(20): p. e618-e651.

2. Lyon, A.R., et al., *Current state of knowledge on Takotsubo syndrome: a Position Statement from the Taskforce on Takotsubo Syndrome of the Heart Failure Association of the European Society of Cardiology.* Eur J Heart Fail, 2016. **18**(1): p. 8-27.

3. Albert, N.M., *Universal Definition and Classification of Heart Failure: New Clarity Brings New Clinical Implications for Health Care Professionals and the Need for New Research.* J Card Fail, 2021. **27**(7): p. 744-746.

4. Ponikowski, P., et al., *2016 ESC Guidelines for the diagnosis and treatment of acute and chronic heart failure: The Task Force for the diagnosis and treatment of acute and chronic heart failure of the European Society of Cardiology (ESC)Developed with the special contribution of the Heart Failure Association (HFA) of the ESC.* Eur Heart J, 2016. **37**(27): p. 2129-2200.

5. Baddour, L.M., et al., *Infective Endocarditis in Adults: Diagnosis, Antimicrobial Therapy, and Management of Complications: A Scientific Statement for Healthcare Professionals From the American Heart Association.* Circulation, 2015. **132**(15): p. 1435-86.

6. Ammirati, E., et al., *Management of Acute Myocarditis and Chronic Inflammatory Cardiomyopathy: An Expert Consensus Document.* Circ Heart Fail, 2020. **13**(11): p. e007405.

7. Spodick, D.H., *Acute pericarditis: current concepts and practice.* JAMA, 2003. **289**(9): p. 1150-3.

8. Force, A.D.T., et al., *Acute respiratory distress syndrome: the Berlin Definition.* JAMA, 2012. **307**(23): p. 2526-33.

9. Australian, P.I.f.t., et al., *Effect of Stress Ulcer Prophylaxis With Proton Pump Inhibitors vs Histamine-2 Receptor Blockers on In-Hospital Mortality Among ICU Patients Receiving Invasive Mechanical Ventilation: The PEPTIC Randomized Clinical Trial.* JAMA, 2020. **323**(7): p. 616-626.

10. Blaser, A.R., et al., *Definition, prevalence, and outcome of feeding intolerance in intensive care: a systematic review and meta-analysis.* Acta Anaesthesiol Scand, 2014. **58**(8): p. 914-22.

11. Kirkpatrick, A.W., et al., *Intra-abdominal hypertension and the abdominal compartment syndrome: updated consensus definitions and clinical practice guidelines from the World Society of the Abdominal Compartment Syndrome.* Intensive Care Med, 2013. **39**(7): p. 1190-206.

12. Patel, A., R.N. Kaleya, and R.J. Sammartano, *Pathophysiology of mesenteric ischemia.* Surg Clin North Am, 1992. **72**(1): p. 31-41.

13. O'Grady, J., *Acute liver failure.* J R Coll Physicians Lond, 1997. **31**(6): p. 603-7.

14. Banks, P.A., et al., *Classification of acute pancreatitis--2012: revision of the Atlanta classification and definitions by international consensus.* Gut, 2013. **62**(1): p. 102-11.

15. European Association for the Study of the, L., *EASL Clinical Practice Guidelines: management of cholestatic liver diseases.* J Hepatol, 2009. **51**(2): p. 237-67.

16. Khwaja, A., *KDIGO clinical practice guidelines for acute kidney injury.* Nephron Clin Pract, 2012. **120**(4): p. c179-84.

17. Chavez, L.O., et al., *Beyond muscle destruction: a systematic review of rhabdomyolysis for clinical practice.* Crit Care, 2016. **20**(1): p. 135.

18. Annane, D., et al., *Guidelines for the Diagnosis and Management of Critical Illness-Related Corticosteroid Insufficiency (CIRCI) in Critically Ill Patients (Part I): Society of Critical Care Medicine (SCCM) and European Society of Intensive Care Medicine (ESICM) 2017.* Crit Care Med, 2017. **45**(12): p. 2078-2088.

19. Spasovski, G., et al., *Clinical practice guideline on diagnosis and treatment of hyponatraemia.* Intensive Care Med, 2014. **40**(3): p. 320-31.

20. Yee, A.H., J.D. Burns, and E.F. Wijdicks, *Cerebral salt wasting: pathophysiology, diagnosis, and treatment.* Neurosurg Clin N Am, 2010. **21**(2): p. 339-52.

21. Robertson, G.L., *Diabetes insipidus: Differential diagnosis and management.* Best Pract Res Clin Endocrinol Metab, 2016. **30**(2): p. 205-18.

22. Fliers, E., et al., *Thyroid function in critically ill patients.* Lancet Diabetes Endocrinol, 2015. **3**(10): p. 816-25.

23. Egi, M., et al., *Hypoglycemia and outcome in critically ill patients.* Mayo Clin Proc, 2010. **85**(3): p. 217-24.

24. Finfer, S., et al., *The NICE-SUGAR (Normoglycaemia in Intensive Care Evaluation and Survival Using Glucose Algorithm Regulation) Study: statistical analysis plan.* Crit Care Resusc, 2009. **11**(1): p. 46-57.

25. Toh, C.H., W.K. Hoots, and S.S.C.o.D.I.C.o.t. ISTH, *The scoring system of the Scientific and Standardisation Committee on Disseminated Intravascular Coagulation of the International Society on Thrombosis and Haemostasis: a 5-year overview.* J Thromb Haemost, 2007. **5**(3): p. 604-6.

26. Subcommittee, A., T. American College of Surgeons' Committee on, and A.w.g. International, *Advanced trauma life support (ATLS(R)): the ninth edition.* J Trauma Acute Care Surg, 2013. **74**(5): p. 1363-6.

27. Warkentin, T.E., *An overview of the heparin-induced thrombocytopenia syndrome.* Semin Thromb Hemost, 2004. **30**(3): p. 273-83.

28. Venkatesan, A., et al., *Case definitions, diagnostic algorithms, and priorities in encephalitis: consensus statement of the international encephalitis consortium.* Clin Infect Dis, 2013. **57**(8): p. 1114-28.

29. Tunkel, A.R., et al., *2017 Infectious Diseases Society of America's Clinical Practice Guidelines for Healthcare-Associated Ventriculitis and Meningitis.* Clin Infect Dis, 2017. **64**(6): p. e34-e65.

30. Horan, T.C., M. Andrus, and M.A. Dudeck, *CDC/NHSN surveillance definition of health care-associated infection and criteria for specific types of infections in the acute care setting.* Am J Infect Control, 2008. **36**(5): p. 309-32.

31. American Thoracic, S. and A. Infectious Diseases Society of, *Guidelines for the management of adults with hospital-acquired, ventilator-associated, and healthcare-associated pneumonia.* Am J Respir Crit Care Med, 2005. **171**(4): p. 388-416.

32. Singer, M., et al., *The Third International Consensus Definitions for Sepsis and Septic Shock (Sepsis-3).* JAMA, 2016. **315**(8): p. 801-10.

33. Edwards, I.R. and J.K. Aronson, *Adverse drug reactions: definitions, diagnosis, and management.* Lancet, 2000. **356**(9237): p. 1255-9.
